# Effects of different polishing techniques on the surface roughness of dental porcelains

**DOI:** 10.1590/S1678-77572010000100004

**Published:** 2010

**Authors:** Işil SARIKAYA, Ahmet Umut GÜLER

**Affiliations:** 1 DDS, PhD, Research Assistant, Department of Prosthodontics, Faculty of Dentistry, Ondokuz Mayis University, Samsun, Turkey.; 2 DDS, PhD, Associate Professor, Department of Prosthodontics, Faculty of Dentistry, Ondokuz Mayis University, Samsun, Turkey.

**Keywords:** Dental porcelain, Polishing techniques, Surface roughness

## Abstract

**Objective:**

The purpose of this study was to evaluate the effects of different polishing techniques on the surface roughness of dental porcelains.

**Material and Methods:**

Fifty-five cylindirical specimens (15x2 mm) were prepared for each feldspathic (Vita VMK 95, Ceramco III) and low-fusing dental porcelain (Matchmaker). Fifty-five specimens of machinable feldspathic porcelain blocks (Vitablocs Mark II), (12x14x18 mm) were cut into 2-mm-thick slices (12x14 mm) with low speed saw. The prepared specimens were divided into 11 groups (n=5) representing different polishing techniques including control ((C) no surface treatment), glaze (G) and other 9 groups that were finished and polished with polishing discs (Sof-Lex) (Sl), two porcelain polishing kits (NTI (Pk), Dialite II (Di)), a diamond polishing paste (Sparkle) (Sp), a zirconium silicate based cleaning and polishing prophy paste (Zircate) (Zr), an aluminum oxide polishing paste (Prisma Gloss) (Pg), and combinations of them. The surface roughness of all groups was measured with a profilometer. The data were analyzed with a 2-way analysis of variance, and the mean values were compared by the Tukey Honestly Significant Difference test (α=0.05).

**Results:**

For all porcelain material groups, the lowest Ra values were observed in Group Gl, Group Sl, Group Pk, and Group Di, which were not significantly different from each other (p>0.05).When comparing the 4 different porcelain materials, the machinable feldspathic porcelain block group (Mark II) demonstrated statistically significantly less Ra values than the other porcelain materials tested (p<0.05). No significant difference was observed between the VMK 95 and Ceramco III porcelain groups (p=0.919), also these groups demonstrated the highest Ra values.

**Conclusion:**

Subjected to surface roughness, the surfaces obtained with polishing and/or cleaning-prophy paste materials used alone were rougher compared to the surfaces finished using Sof-lex, Dialite, and NTI polishing kit. Polishing kits and discs were found more effective than the polishing pastes used alone or combined use with Sof-lex discs, thus improving surface smoothness.

## INTRODUCTION

In addition to the improved esthetic properties, such as translucency, color and intensity, the main advantages of dental porcelain materials are excellent biocompatibility and durability^3^.

Although occlusal adjustment of porcelain restorations may be necessary for correction of inadequate contours or improved esthetics, roughened ceramic surface creating with this procedure, which may cause an increased rate of plaque accumulation, gingival inflammation and adverse soft tissue reaction^1,10,20,24^. In addition porcelain reduction with grinding may also cause decrease the strength of the ceramic restoration^4,12,14^.

Since the final occlusal adjustments of a ceramic restoration has to be made after cementation, there is always need for a careful intraoral polishing of the surfaces. Exposed porcelain surfaces should be smoothed to prevent excessive wear of the opposing dentition and to minimize plaque retention^18^. Highly polished porcelain surfaces have strength values comparable to those of specimens that were polished and glazed^3^. However, chairside porcelain polishing is efficient and easy for the clinician. Intraoral polishing also provides infection control by eliminating repeated laboratory procedures.

Surface roughness (Ra) refers to the finer irregularities of the surface texture that usually result from the action of the production process or material condition and is measured in micrometers (µm)^16^. Generally, a smooth surface is desirable to reduce retention of bacteria and to have a shiny appearance^7^. Various studies are available to support using different polishing techniques on porcelain surface instead of glazing^2,1,17,18,24^.

The purpose of this study was to evaluate the effects of different polishing techniques on the surface roughness of different dental porcelains. The hypothesis for this study was that the different polishing techniques would have different effects on the surface roughness of dental porcelains.

## MATERIAL AND METHODS

In the present study, four commonly used and commercially available dental porcelains and different polishing systems were investigated in this study (Figure 1). Fifty-five cylindrical specimens (15x2mm) were prepared for each of feldspathic and low-fusing dental porcelains by one investigator who condensed the porcelains into a polyvinylsiloxane mold in a standardized manner^6^. After each specimen was mixed using the same amount of porcelain and liquid, placed into the mold and compressed with a plastic plunger. The excess moisture was absorbed by using a tissue (Selpak; Eczaclbasl Group, Kocaeli, Turkey). After removal from the mold, the specimens were fired in one furnace (Programat P80; Ivoclar-Vivadent, Liechtenstein) according to the manufacturer’s directions (approximately 920-960°C).

**Figure 1:** Materials used in this study

**Material Type**	**Product**	**Manufacturer**
Feldspathic porcelain	VMK 95	Vita Zahnfabrik, Germany
Feldspathic porcelain	Ceramco III	Degudent GmbH, USA
Low-fusing porcelain	Matchmaker MC	Schottlander, UK
Feldspathic porcelain blocks	Vitablocs Mark II	Vita Zahnfabrik, Germany
Finishing and polishing disc	Sof- Lex	3M ESPE, USA
Porcelain polishing kit	NTI CeraGlaze	NTI- Kahla GmbH, Germany
Porcelain polishing kit	Dialite II	Brasseler, USA
Diamond polishing paste	Sparkle	Pulpdent, USA
Zirconium silicate cleaning- polishing prophy paste	Zircate	Dentsply Int. Inc., USA
Aluminum oxide polishing paste	Prisma Gloss	Dentsply Int. Inc., USA

Fifty-five specimens of machinable ceramic blocks (12x14x18mm) were cut into 2-mm-thick slices (2x14 mm) with a low-speed saw (Isomet Low Speed Saw, Buehler, Lake Bluff, IL, USA). All ceramic discs were wet-ground with 600-grit silicon carbide paper during 10 s on a 300-rpm grinding machine (Buehler Metaserv, Buehler, Germany).

The prepared specimens were randomly divided into 11 groups of 5 specimens each according to the polishing techniques. The polishing procedure was done by a single investigator and different polishing groups are listed in Figure 2. Group C - specimens served as the control group with no polishing procedure applied. Group Gl - specimens were glazed using the specific glaze medium for each case. Group Sl - specimens were polished with a series of 12.7-mm-diameter polishing discs (Sof-Lex; 3M/ ESPE, St. Paul, MN, USA) mounted on an electric handpiece set at a speed of 10,000 rpm during 10 s for coarse and medium discs, and 30,000 rpm during 10 s for fine and superfine discs, according to the manufacturer’s instructions. Group Pk - specimens were polished with NTI Cera Glaze polishing kit on an electric handpiece at 15,000 rpm for 10 s with pre-polishing wheel, at 10,000 rpm during 10 s with refined finishing wheel and at 5,000 rpm for 10 s with high-shine polishing wheel according to the manufacturer’s instructions. Group Di - specimens were polished with Dialite II porcelain polishing kit including pre, fine and high-shine wheels on an electric handpiece at 10,000 rpm for 10 s following the manufacturer’s instructions. Groups Sp, Zr and Pg - Sparkle diamond polishing paste, Zircate zirconium silicate cleaning-prophy paste and Prisma Gloss aluminum oxide polishing paste, respectively, were applied for 10 s to the specimens with a prophylaxis rubber cup (Kenda Polishers, Kenda AG, Liechtenstein) mounted on an electric handpiece at 15,000 rpm. Group specimens were applied with a prophylaxis rubber cup on an electric handpiece at 15,000 rpm for 10 s. Group SlSp - specimens were polished as in Group Sl and diamond polishing paste was applied as described for Group Sp. Group SlZr specimens were polished as in Group Sl and zirconium silicate cleaning-prophy paste was applied as described for Group Zr. Group SlPg specimens were polished as in Group Sl and aluminum oxide polishing paste was applied as described for Group Pg. The specimens were then ultrasonically cleaned (Eurosonic Energy, Euronda, Italy) with deionized water for 10 min and dried.

**Figure 2:** Polishing methods

**Group**	**Polishing techniques**
Group- C	Control (no surface treatment)
Group- Gl	Glaze
Group- Sl	Sof- Lex
Group- Pk	NTI CeraGlaze Polishing kit
Group- Di	Dialite II
Group- Sp	Sparkle
Group- Zr	Zircate
Group- Pg	Prisma Gloss
Group- SlSp	Sof- Lex+ Sparkle
Group- SlZr	Sof- Lex+ Zircate
Group- SlPg	Sof- Lex+ Prisma Gloss

The specimens were stabilized with silicone impression material into a brass mold and three roughness measurements (Ra, µm) were performed on each sample using a profilometer (Perthometer M2, Mahr GmbH, Germany). A cutoff value of 0.25 mm allowed detecting those irregularities^8,22^. A diamond stylus (NHT-6) of 2 µm radius and 90° stylus angle was traversed at a constant speed across each of the finished samples of ceramic sample with a force of 0.7 N. Before measurements of each group, the profilometer was calibrated. All profilometer records were made as close as possible to the sample center^8^. For each specimen, 3 measurements were made and the mean was calculated to obtain the general surface characteristics of the specimens. The Ra value describes the mean value for a surface that has been traced by the profilometer^8,20^. A lower Ra value indicates a smoother surface.

The effect of porcelain type and polishing procedure as well as their interactions on the surface roughness was evaluated by two-way ANOVA tests using SPSS for Windows statistical software version (12.0.1; SPSS Inc, Chicago, IL, USA). The mean values were than compared by the Tukey honestly significant difference test (α=0.05).

## RESULTS

According to the two-way ANOVA results, porcelain materials, polishing techniques, and their interaction were statistically significant (p<0.05) (Table 1). Mean surface roughness values (and standard deviation) and group differences (Ra) of the feldspathic porcelain materials (Vita VMK 95, Ceramco III), low-fusing porcelain material (Matchmaker MC), and machinable feldspathic porcelain block (Vitablocs Mark II) are listed in Tables 2, 3, 4 and 5, respectively.

**Table 1:** Two-way ANOVA for porcelain materials and different polishing techniques

**Variable (source)**	***df***	**Sum of squares**	**Mean squares**	**F**	**P**
Polishing technique	10	25.328	2.533	35.107	.000*
Porcelain	3	31.779	10.593	146.830	.000*
Interaction	30	17.431	.581	8.054	.000*
Error	176	12.698	.072		

* Significant difference at p<.05.

**Table 2:** Mean (SD) of surface roughness and differences between groups for Vita VMK 95 porcelain

**Group**	**Ra**	Difference*
C	1.134 (0.64)	a,b
Gl	0.724 (0.19)	a
Sl	0.729 (0.17)	a
Pk	0.640 (0.15)	a
Di	0.700 (0.23)	a
Sp	1.639 (0.26)	b,c
Zr	2.241 (0.41)	c
Pg	1.728 (0.47)	b,c
SlSp	1.929 (0.76)	b,c
SlZr	1.731 (0.48)	b,c
SlPg	1.122 (0.24)	a,b

* Different letters indicate statistically significant difference between the groups (p<0.05).

**Table 3:** Mean (SD) of surface roughness and differences between groups for Ceramco III porcelain

**Group**	**Ra**	Difference*
C	1.667 (0.21)	c,d
Gl	0.609 (0.23)	a
Sl	0.617 (0.10)	a
Pk	0.639 (0.10)	a
Di	0.999 (0.26)	a,b
Sp	1.824 (0.39)	c,d
Zr	1.974 (0.12)	d
Pg	1.872 (0.18)	c,d
SlSp	1.400 (0.19)	b,c
SlZr	1.481 (0.22)	b,c
SlPg	1.595 (0.29)	c,d

* Different letters indicate statistically significant difference between the groups (p<0.05).

**Table 4:** Mean (SD) of surface roughness and differences between groups for Matchmaker MC porcelain

**Group**	**Ra**	Difference*
C	1.015 (0.23)	b,c,d
Gl	0.456 (0.11)	a
Sl	0.757 (0.11)	a,b,c
Pk	0.691 (0.16)	a,b
Di	0.366 (0.08)	a
Sp	0.746 (0.26	a,b,c
Zr	1.728 (0.20)	f
Pg	1.700 (0.10)	f
SISp	1.262 (0.32)	d,e,f
SIZr	1.652 (0.17)	e,f
SIPg	1.175 (0.41)	c,d,e

* Different letters indicate statistically significant difference between the groups (p<0.05).

**Table 5:** Mean (SD) of surface roughness and differences between groups for Mark II porcelain

**Group**	**Ra**	Difference*
C	0.314(0.12)	a
Gl	0.406(0.18)	a,b
Sl	0.431(0.09)	a,b
Pk	0.584(0.04)	b
Di	0.434(0.04)	a,b
Sp	0.380(0.12)	a,b
Zr	0.312(0.11)	a
Pg	0.342(0.07)	a
SlSp	0.386(0.06)	a,b
SlZr	0.321(0.12)	a
SlPg	0.364(0.13)	a,b

* Different letters indicate statistically significant difference between the groups (p<0.05).

For the VMK 95 feldspathic porcelain material, the lowest Ra values were observed in Group Gl (0.724), Group Sl (0.729), Group Pk (0.640), Group Di (0.700), Group C (1.134) and Group SlPg (1.122), which were not significantly different from each other (p=0.732). The highest Ra value in the VMK 95 porcelain material was observed in Group Zr (2.241), which were not significantly different from Group Sp (1.639), Group Pg (1.728), Group SlSp (1.929) and Group SlZr (1.731) (p=0.466). The differences between VMK 95 feldspathic porcelain material group are listed in Table 2. For the Ceramco III feldspathic porcelain material, the lowest Ra values were observed in Group Gl (0.609), Group Sl (0.617), Group Pk (0.639) and Group Di (0.999), which were not significantly different from each other (p=0.230). The highest Ra value in the Ceramco III feldspathic porcelain material was observed in Group Zr (1.974) which were not significantly different from Group C (1.667), Group Sp (1.824), Group Pg (1.872) and Group SlPg (1.595) (p=0.265) (Table 3).

For the low-fusing porcelain material, the lowest Ra values were observed in Group Di (0.366), which were not significantly different from Group Gl (0.456), Group Sl (0.757), Group Pk (0.691) and Group Sp (0.746) (p=0.203). No significant difference was observed among Group SlZr (1.652), Group SlPg (1.175), Group SlSp (1.262), Group Pg (1.700) and Group Zr (1.728), also these groups demonstrated the highest Ra values for the low-fusing porcelain material tested (p=0.063) (Table 4).

For the machinable feldspathic porcelain block, the lowest Ra values were observed in Group C (0.314), Group Gl (0.406), Group Sl (0.431), Group Di (0.434), Group Sp (0.380), Group Zr (0.312), Group Pg (0.342), Group SlSp (0.386), Group SlZr (0.321) and Group SlPg (0.364), which were not significantly different from each other (p=0.816). Group Pk (0.584) differed significantly from these groups (p<0.05). No significant difference was observed among Group Pk (0.584), Group Gl (0.406), Group Sl (0.431), Group Di (0.434), Group Sp (0.380), Group SlSp (0.386) and Group SlPg (0.364) (p=0.100) (Table 5).

When comparing the 4 different porcelain materials, the machinable feldspathic porcelain block (Mark II) demonstrated significantly lower Ra values than the other porcelain materials tested (p<0.05). No significant difference was observed between the VMK 95 and Ceramco III porcelains (p=0.919), which presented the highest Ra values.

## DISCUSSION

The hypothesis set as the premise of this study was accepted, since different polishing techniques affected the surface roughness of the evaluated dental porcelains. In the present study the efficiency of different porcelain polishing kits, polishing discs, cleaning-prophy paste materials and their combinations were compared. These materials were selected among frequently used dental porcelain systems as being fast and effective on creating smoothed porcelain surfaces.

The Ra parameter obtained with a profilometer is used to describe the surface texture of the porcelain specimens. This parameter describes the overall roughness of a surface and can be defined as the arithmetical average value of all absolute distances of the roughness profile from the center line within the measuring length^23^.

Various finishing and polishing techniques can use on porcelain surface to preserve its structural resistance and obtain a clinically acceptable smoothness comparing with glazing^1,2,11,17,18,24^. Previous studies on surface roughness of dental porcelains demonstrated that very smooth surfaces were obtained when restorations were polished with rubber wheel, Sof-lex discs, porcelain polishing kit, diamond paste and aluminum oxide paste^1,5,17-19,21,24^. In this study, it was observed that using porcelain polishing disc or polishing kits, diamond pastes or alumina pastes alone created surfaces as smooth as glazed specimens as similar studies. This result indicates that polishing kits and disc systems had a similar effect on ceramic surface roughness compared to glazing.

According to Al Wahadni, et al.^1^(1998), polishing of a ceramic restoration by diamond paste at the final stage after clinical adjustment was equivalent to reglazing. Hulterstrom, et al.^9^(1993) found that Sof-lex system produced the best results for Mark I porcelain when no polishing paste was used. In the present study, the Soflex system produced the lowest Ra values for all porcelains. Final polishing with a diamond-containing polishing paste after polishing with the Sof-lex system produced no significant decrease in the Ra value for Mark II ceramics. Hulterstrom, et al^9^ (1993) recommended that final polishing with the fairly expensive diamond paste did not significantly improve the smoothness of the ceramic surface polished with the Sof-lex system. Furthermore several studies have shown that final polishing with a diamond paste did not improve the surface smoothness of ceramic restorations^8,9,12^.

Due to the difficult in reaching intraoral access, occlusal corrections may result in insufficient polishing and the formed microcracks may be susceptible to later catastrophic fractures. Therefore, much care must be taken in order to make a careful polishing of inlay areas that had been previously subjected to rotary occlusal corrections to prevent from this particular problem^13^.

In the present study, the surfaces obtained with polishing and/or cleaning-prophy paste materials were rougher when used alone compared with the surfaces finished through using Sof-lex, Dialite, and NTI polishing kit. This finding is in agreement with previous reports investigating the effects of different polishing systems on the surface roughness of ceramics^8,9,15,19^.

According to the results of present study, the machinable feldspathic porcelain block group (Mark II) demonstrated significantly lower Ra values than the other porcelain materials. No significant difference was observed between the VMK 95 and Ceramco III porcelains, which presented the highest Ra values. Several studies on finishing and polishing of dental ceramics have been published, but there is a lack of studies investigating the effectiveness of different polishing techniques for the newer types of dental porcelain, such as Mark II porcelain blocks. There were not significant differences among the various polishing techniques for Mark II porcelains, and this is thought to be due to the extreme hardness of Mark II feldspathic blocs.

The present study has some limitations. Although intraoral polishing systems have been used, this is an in vitro study and so the efficiency of polishing systems might be different under clinical conditions. Furthermore, different results might be expected with different types of porcelain and polishing protocols. Further investigations are necessary to evaluate the surface roughness of other porcelain systems after polishing with different polishing protocols.

## CONCLUSION

Within the limitation of this study, glazing and polishing discs and kits have shown lower Ra values than those obtained using polishing pastes. Polishing kits and discs were found more effective than the polishing pastes used alone or combined with Sof-lex discs, resulting in improved surface smoothness. If occlusal adjustment of a ceramic restoration has to be made after cementation there is always need for a careful intraoral polishing with polishing kits and discs.
